# Effect of Post-Heat Treatment Temperature on Interfacial Mechanical Properties of Cold-Rolled Ti/Al Clad Material

**DOI:** 10.3390/ma15176103

**Published:** 2022-09-02

**Authors:** Sang-Kyu Yoo, Ji-Won Kim, Myung-Hoon Oh, In-Chul Choi

**Affiliations:** School of Materials Science and Engineering, Kumoh National Institute of Technology, Gumi 39177, Korea

**Keywords:** Ti/Al-clad material, post heat treatment, bonding interface, mechanical properties, nanoindentation

## Abstract

Titanium and titanium alloys possess low density, high specific strength, and excellent corrosion resistance, but are expensive and have low formability at room temperature. Therefore, to reduce cost and achieve excellent properties, titanium and titanium alloys are jointed with aluminum and its alloys, which are inexpensive and have low density and excellent room temperature formability. Cladding is a widely used solid-state bonding technique, and the post-heat treatment of titanium/aluminum clad materials is required to improve their interfacial properties, which is important to ensure the reliability of Ti/Al-clad materials. The interfacial properties of Ti/Al-clad materials are significantly affected by changes in the microstructure and mechanical properties after the post-heat treatment. Thus, in this study, the relationship between the microstructure and mechanical properties at the interface of Ti/Al-clad materials was analyzed after the post-heat treatment at several different temperatures. The thick diffusion and intermetallic compound layer was formed with post-heat treatment owing to the active diffusion of Al atoms. As a result, their uniaxial and nanomechanical properties were varied with the interfacial characteristics of the Ti/Al-clad material.

## 1. Introduction

Titanium and titanium alloys are excellent materials for the automobile and aerospace industries, because of their low density, high specific strength, and excellent corrosion resistance. However, owing to their high price and low formability at room temperature, the application scope of titanium and titanium alloys is limited [1,2,3]. To overcome these issues, heterogeneously bonded composite materials fabricated using solid-state bonding techniques have been widely adopted in various industries [4,5,6]. Particularly, joining titanium and titanium alloys with aluminum or its alloys, which are inexpensive, have low density and excellent room temperature formability, can result in a composite with lower cost and better room temperature formability compared to titanium alloys [7,8,9].

Solid-state bonding techniques are widely used in the industry to fabricate composite metals. Multiple types of solid-state bonding techniques are known, including cladding, vacuum brazing, and friction welding [10]. Among them, cladding produces composite materials that retain excellently the properties of all component materials, and have a microstructurally continuous interface formed through thermally activated diffusion [11]. This process is also economically efficient and easily automatable for the continuous production of clad materials [12,13]. However, during the cladding process, the mechanical properties of the bonded interface are significantly affected by phase transformation and microstructural changes at the bonded interface. In addition, because cladding is a mechanical bonding process, the resulting bonding strength is relatively low compared with other cladding processes, because of insufficient atomic diffusion at the bonded interface [14]. Delamination occurs easily in Ti/Al-clad materials, resulting in a relatively low elongation at break [15]. Therefore, the post-heat treatment of Ti/Al-clad materials might be required to increase their bonding strength and elongation at break [16,17,18].

To ensure the reliability of the Ti/Al-clad materials, the relationship between the microstructural and mechanical properties of the bonding interface after post-heat treatment must be elucidated. Thus, the purpose of this study is to understand the effect of post-heat treatment on the interfacial mechanical behavior of Ti/Al-clad materials. As a first step, Ti/Al-clad plates were manufactured by a rolling process, and then the post-heat treatments were preformed to enhance the interfacial mechanical properties by the interfacial diffusion process. Then, the microstructural changes in the bonding interface at different post-heat treatment temperatures were observed, and the general mechanical properties were estimated using a commercial uniaxial tensile test. In addition, nanoindentation tests are one of the ways to characterize the interfacial mechanical properties of materials that can be applied to localized areas, such as the Ti/Al interface [19,20,21]. That is, hardness (*H*) and reduced modulus (*E*_r_) at the Ti/Al interface can be obtained from the load (*P*)–displacement (*h*) curves without additional characterization. Finally, the relationship between the microstructure and the interfacial mechanical properties measured through nanoindentation tests was analyzed.

## 2. Materials and Methods

**Figure 1:**
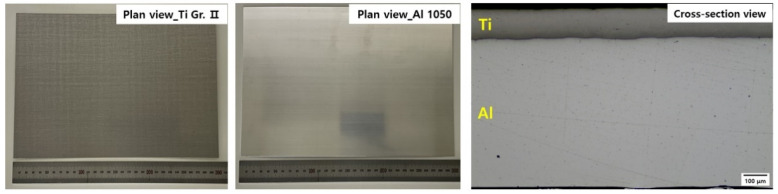
The part of the as-rolled Ti/Al-cladding materials: (**left**) the plan view of Ti side, (**middle**) the plan view of Al side, and (**right**) the cross-section view.

In this study, Ti Grade II and Al 1050 sheets were used for the manufacturing of the clad material; the composition of each material is listed in Table 1. The Ti/Al-clad materials were prepared via roll bonding by Korea Clad Tech., Daegu, South Korea. The initial thicknesses of the Ti coil and Al coil were 0.15 mm and 1 mm, respectively. Before performing roll bonding, the surfaces of Ti and Al were scribed mechanically. In the roll bonding process at 400 °C, the reduction ratio was higher than 25%. After the processing, the total thickness of the clad materials was 0.8 mm, with the thicknesses of Ti Grade II and Al 1050 being 0.13 and 0.67 mm, respectively. This means that the deformation degree of Ti and Al is not same. That is, the deformation degree of the Al coil is higher than that of the Ti coil. part of the final Ti/Al-cladded product is shown in Figure 1.

The Ti/Al-clad materials were post-heat-treated in a quartz-tube furnace (M-13P-70, HANTECH, Gunpo, South Korea) at 300, 400, 500, 600, and 650 °C in AN Ar atmosphere at a heating/cooling rate of 5 °C/min and a holding time of 30 min at each maximum temperature. To observe the microstructural change in the cross-sectional area of the Ti/Al-clad materials, the Ti/Al-clad specimens were vertically mounted using a hot mounting machine (ETOS-100, MTDI, Deajeon, South Korea) with an acrylic resin. The samples were mechanically ground using silicon carbide paper and polished using diamond suspensions. Then, the microstructure near the Ti/Al interface was observed using optical microscopy (ECLIPSE L150, Nikon, Tokyo, Japan) and field emission scanning electron microscopy (MAIA III, TESCAN Ltd., Brno, Czech Republic). To observe the microstructures of Ti and Al, the Ti/Al-clad material was etched with 10 mL hydrofluoric acid and 100 mL distilled water. Qualitative compositional and phase analyses were performed using energy dispersive spectroscopy (EDS, AZtec Energy Advanced Package, Oxford Ins., Abingdon, UK). To identify the presence of intermetallic compounds at the interface, X-ray diffraction patterns were measured using a MiniFlex 600 (Rigaku, Tokyo Japan). XRD patterns were obtained via Cu-Kα radiation with the step time of 5°/min. Uniaxial tensile and nanoindentation tests were performed to analyze the mechanical properties of the Ti/Al-clad materials with and without post-heat-treatment. For the uniaxial tensile tests, sub-sized specimens with a gauge length of 25 mm, width of 6 mm, and thickness of 0.8 mm in the direction perpendicular to the rolling direction were used. Tensile tests were conducted at a strain rate of 0.001/s at room temperature using a universal testing machine (MINOS-100, MTDI, Daejeon, South Korea) with an extensometer. Nanoindentation tests were performed to evaluate the nanohardness and modulus of the Ti and Al base materials and bonding interface after the post-heat treatment. The samples were mechanically ground using silicon carbide paper and polished carefully using diamond suspensions to remove the undesired deformed layer. For the nanoindention test, a nanoindenter (i-Micro nanoindenter, KLA Co., Milpitas, CA, USA) was used with a three-sided pyramidal Berkovich diamond indenter. The hardness values were determined from the measured load divided by the projected tip area at the penetration depth of the Berkovich indenter into the sample. The elastic modulus (*E*) was calculated according to $E=\left\{\left(1-\nu^{2}\right)\cdot E_{i}\cdot E_{r}\right\}/\left\{E_{i}-\left(1-\nu_{i}^{2}\right)E_{r}\right\}$ where ν is the Poisson’s ratio of the specimen, $E_{r}$ is the reduced modulus measured from nanoindentation, and $E_{i}$ and $\nu_{i}$ are the elastic modulus and Poisson’s ratio of indenters, respectively. The correction of the load frame stiffness and the determination of the tip area function were conducted according to the process suggested by Oliver and Pharr [22]. During the nanoindentation test, the Berkovich indenter penetrates into the sample surface until the maximum indentation depth of 1 μm. The indentation strain rate ((d*P*/d*t*)/*P* = 2(d*h*/d*t*)/*h*) is 0.05/s, and lthe oad holding time at the maximum load is 1 s. The thermal drift of the system was less than ±0.05 nm/s to minimize the effect.

## 3. Results and Discussion

**Figure 2:**
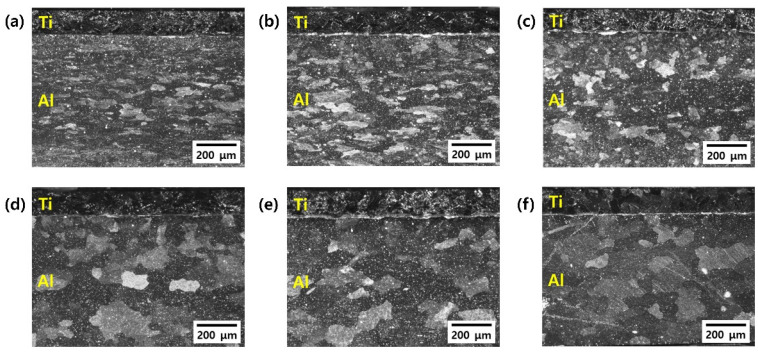
OM images of the Ti/Al-clad materials after post-heat treatment for 30 min at different temperatures: (**a**) As-rolled, (**b**) 300 °C, (**c**) 400 °C, (**d**) 500 °C, (**e**) 600 °C, and (**f**) 650 °C.

**Figure 3:**
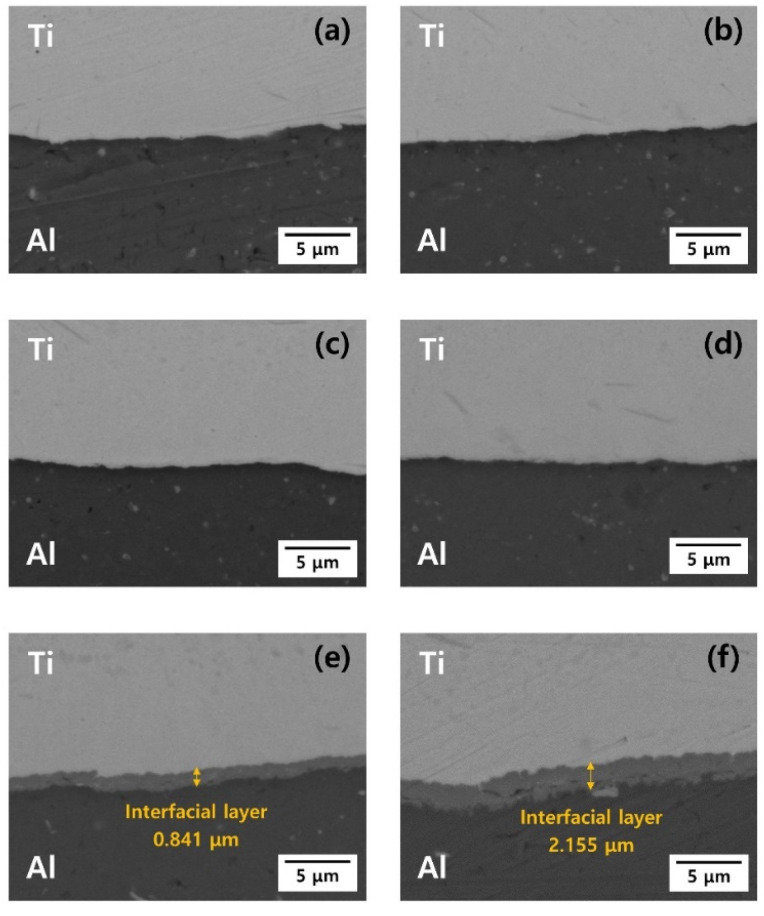
Backscattered electron images of the Ti/Al-clad interface after post-heat treatment for 30 min at different temperatures: (**a**) As-rolled, (**b**) 300 °C, (**c**) 400 °C, (**d**) 500 °C, (**e**) 600 °C, and (**f**) 650 °C.

**Figure 4:**
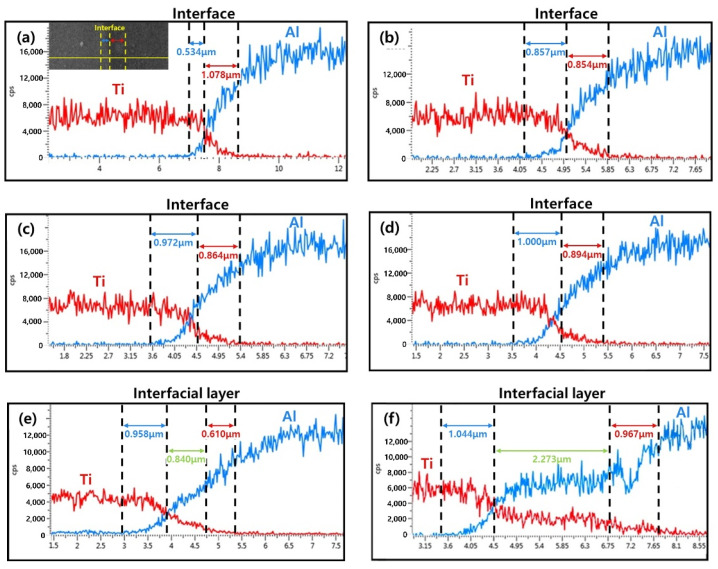
Composition analysis of the Ti/Al-clad interface by energy dispersive spectroscopy (EDS), which shows the variation in the thickness of the diffusion layer after post-heat treatment for 30 min at different temperatures: (**a**) as-rolled, (**b**) 300 °C, (**c**) 400 °C, (**d**) 500 °C, (**e**) 600 °C, and (**f**) 650 °C.

Figure 2 shows OM images of the Ti/Al-clad materials before and after the post-heat treatment. Before the post-heat treatment, the Ti grains were equiaxed, but the Al grains were small and elongated. There was almost no change in Ti grains because the temperature range of the heat treatment was not sufficient to evolve the microstructure of Ti with a relatively high melting point. However, the deformed grains of Al were recovered by increasing the temperature of the post-heat treatment. That is, after the heat treatment, the grain sizes increased and the shapes of the Al grains became more equiaxed. Figure 3a–f show backscattered electron (BSE) images of the Ti/Al-clad materials before and after the post-heat treatment at 300, 400, 500, 600, and 650 °C for 30 min. In the as-clad material, there are no new phases at the Ti/Al interface; essentially, any microstructural changes in the interfacial area of the Ti/Al-clad material can hardly be observed after mechanical rolling. Furthermore, even in the samples post-heat-treated at 300, 400, and 500 °C, no significant microstructural changes were observed at the Ti/Al interface. However, a new layer with a thickness of ~0.841 μm was formed at the Ti/Al interface in the sample post-heat-treated at 600 °C. In the sample post-heat-treated at 650 °C, this layer was already ~2.155 μm thick. These results show that sufficient activation energy (or thermal energy; in this case, a temperature of 600 °C or higher) is required to form a new phase layer. To qualitatively analyze either the interface layer or the new phase layer that can be formed during rolling and post-heat treatment, line analysis was performed using EDS, and the results are shown in Figure 4. In contrast to the BSE images, all the samples show an atomic diffusion layer around the Ti/Al interface.

To clarify the evolution of the diffusion layer at the Ti/Al interface, the diffusion coefficients for Al and Ti may be considered, which are usually found using an Arrhenius type equation:

(1) $$D=D_{0}\exp\left(-Q/RT\right)$$

where *D*_0_ is the pre-exponential parameter, *Q* is the activation energy of diffusion, *R* is the gas constant, and *T* is the absolute temperature [23,24]. The values of *D*_0_ and *Q* depend on temperature, and are summarized in Table 2. The diffusion distance is proportional to $\sqrt{Dt}$, where *t* is time. That is, the diffusion distance varies with the post-heat treatment temperature and time. Therefore, it can be predicted that the diffusion distance increases with an increasing post-heat treatment temperature, which is in good agreement with the EDS results shown in Figure 4. In addition, in the post-heat treatment temperature range shown in Figure 4, the diffusion distance of Al from the interface is larger than that of Ti because the diffusion coefficient of Al in Ti is several orders of magnitude higher than that of Ti in the Al matrix. While at 300–500 °C, the diffusion layer is observed (Figure 4a–d), its thickness rapidly increases above 600 °C owing to the exponential increase in the diffusion coefficients of Al and Ti.

**Figure 5:**
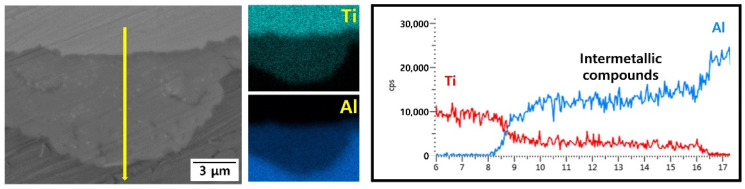
EDS analysis of the specimens post-heat treatment at 650 °C.

**Figure 6:**
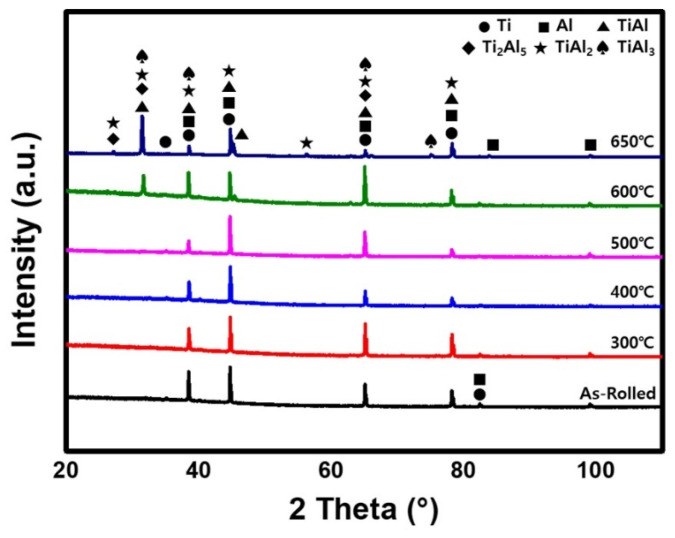
XRD patterns of Ti/Al-clad materials: (black) as-rolled, (red) 300 °C, (blue) 400 °C, (pink) 500 °C, (green) 600 °C, and (dark blue) 650 °C.

**Figure 7:**
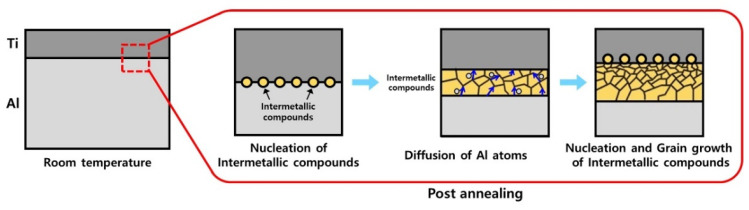
Formation mechanism of intermetallic compounds during post-heat treatment.

Figure 5 shows the EDS results for the Ti/Al bonding interface of the specimens post-heat-treated at 650 °C, which is also observed at the interface of the post-heat-treated specimens at 600 °C. In contrast to Figure 4a–d, where the contents of Ti and Al change gradually, in Figure 5, there is a region in which the ratio of Ti and Al is constant. According to the quantitative analysis of the region, the layer consists of 24.70 at. % Ti and 75.30 at. % Al, which are the average compositions in the measured area. Therefore, to identify the phase of the newly formed layer at the Ti/Al interface during post-heat treatment at different temperatures, the X-ray diffraction (XRD) patterns were measured, and the results are shown in Figure 6. Before heat treatment, there are only Ti and Al peaks in the XRD patterns. In addition, there are no intermetallic compounds peaks in the XRD patterns of post-heat-treated samples at 300 °C, 400 °C, and 500 °C. However, the post-heat treatment specimens at 600 °C and 650 °C show peaks of TiAl, TiAl_2_, Ti_2_Al_5_, and TiAl_3_ intermetallic compounds. Usually, various intermetallic compounds, such as Ti_3_Al, TiAl, TiAl_2_, Ti_2_Al_5_, and TiAl_3_, are formed through different metallic bonding processes at the Ti/Al interface. In this study, it is possible to observe four different intermetallic compounds at the Ti/Al interface. According to Xu et al.’s result on the effective heat for the formation of intermetallic compounds [28], TiAl_3_ has the lowest formation free energy, and thus may preferably form at the Ti/Al interface during the post-heat treatment of clad materials, which is consistent with previous studies [28,29,30]. Then, the next possible intermetallic compound may be TiAl, which has a low formation energy and can be formed by the reaction between TiAl_3_ and Ti [28]. In addition, TiAl_2_ and Ti_2_Al_5_ can be formed in a series of solid-state reactions (such as diffusion in the solid phase) from TiAl at relatively low temperatures [31]. Therefore, the relative intensities of the TiAl, TiAl_2_ and Ti_2_Al_5_ peaks in the XRD patterns are higher than that of TiAl_3_, because the TiAl_3_ phase can be consumed to form TiAl, TiAl_2_ and Ti_2_Al_5_ intermetallic compounds. However, as show in Figure 6, Ti_3_Al peaks were not observed in the measured XRD patterns. This is related to the highest heat of formation of Ti_3_Al [28]. The formation mechanism of the intermetallic compound layer during the post-heat treatment is shown in Figure 7; the intermetallic compounds’ nuclei are first generated at the Ti/Al interface, and then the diffusion of Al atoms through the grain boundaries of intermetallic compounds increases the thickness of the intermetallic compound layer. Thus, as shown in Figure 4e,f, the diffusion of Al and Ti becomes more active with increasing post-heat treatment temperature and time, such that the final thickness of the intermetallic compound layer increases, which is also in good agreement with the literature [32,33,34].

**Figure 8:**
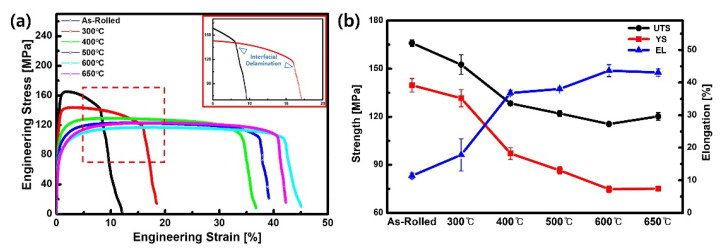
Room temperature tensile properties before and after post-heat treatment at different temperatures: (**a**) stress–strain curves and (**b**) ultimate tensile strength, yield strength, and elongation.

Tensile tests at room temperature were performed to measure the mechanical properties of the Ti/Al-clad materials. Figure 8a shows the stress–strain curves of the Ti/Al-clad materials with and without post-heat treatment. The determined yield strength, ultimate tensile strength, and elongation at break for each specimen are summarized in Figure 8b and Table 3. The as-clad specimen exhibits the highest ultimate tensile strength of 166 MPa and the lowest elongation at break of 11%. Usually, the mechanical properties of the clad materials are greatly affected by the properties and volume fractions of the constituent materials [16,35]. During cladding with a 25% reduction ratio at 400 °C, the strengths of the Ti and Al are increased by the strain hardening effect. According to the literature, the yield strength of Ti Grade II is increased to 304 MPa under a reduction ratio of 56%, and the yield strength of Al 1050 is increased to 145 MPa under a reduction ratio of 75% [36,37]. In addition, the elongation at break of Ti and Al is changed by the strain hardening effect, with values of 33% and 7% [36,37]. Then, a simple rule of mixtures can be adopted to predict the yield strength and elongation at break of the Ti/Al-clad material. The calculated yield strength and elongation at break of the as-rolled specimen are approximately 170.8 MPa and 11.2%, respectively, which is in good agreement with the experimentally measured values in this study.

With an increasing post-heat treatment temperature from 300 to 600 °C, the yield strength and ultimate tensile strength of the Ti/Al-clad material decrease from 131.6 MPa and 152.6 MPa to 74.7 MPa and 115.5 MPa, respectively. In contrast to the strength, the elongation at break increases from 17.8 to 43.6%. These tendencies have been attributed to the general heat treatment effect: dislocation rearrangement and recrystallization because of the recovery of Al, which occupies a relatively large area in the Ti/Al-clad material [38]. In addition, the texture may also be important for understanding the mechanical behaviors of materials, whose characterization is beyond the scope of our study. However, an analysis that combines the evolution of the texture with the mechanical behavior of the clad material is desirable in future.

With the increase in post-heat treatment temperature from 600 to 650 °C, the elongation at break slightly decreases to 43.1%, while the strength increases to 120.3 MPa. As previously shown in Figure 5; Figure 6, the intermetallic compound layer is considered to form at the interface of the Ti/Al-clad material at 600 °C, because it is not observed in the samples post-heat-treated at 300–500 °C. In addition, no phase transformations of Ti and Al occur at the post-heat treatment temperatures. In general, the intermetallic compound layer at the interface of dissimilarly jointed materials can improve the interfacial strength, but reduce the interfacial properties, owing to its brittleness [39,40]. Therefore, there is a critical thickness of the intermetallic compound layer, which is sufficient to ensure proper bonding properties while being not too large to degrade the mechanical properties of the dissimilarly jointed material [41]. This intermetallic compound layer at the interface acts as a stress concentration area, and reduces the elongation at break because of the formation of voids and cracks at the interface [30,32].

With increasing the post-heat treatment temperature, the microstructures of Ti and Al are recovered from the work-hardened state that is induced during the cladding process (see Figure 2). This recovery alters the plastic deformation behaviors, such as strength and strain-hardening exponent, of Ti and Al. As shown in Figure 8b and Table 3, the yield strength is reduced with an increase in the post-heat treatment temperature. According to Holloman’s equation [42], the strain-hardening exponent can be obtained from the slope of the log(true stress)–log(true strain) curves. The estimated values of the strain-hardening exponent are increased from 0.102 for 400 °C to 0.168 for 650 °C. As a result, in the stress–strain curves of Ti/Al-clad specimens heat-treated at 400 °C, 500 °C, 600 °C, and 650 °C, the engineering stress seems to overlap in the engineering strain range of 25–35%.

**Figure 9:**
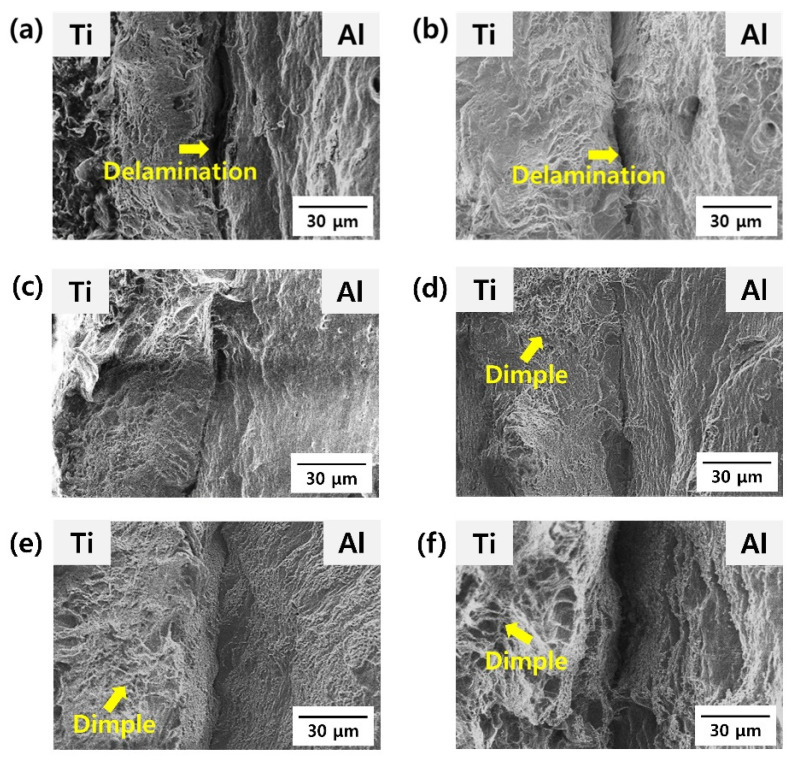
Fracture surface after the tensile tests of the specimens post-heat-treated at different temperatures: (**a**) as-rolled, (**b**) 300 °C, (**c**) 400 °C, (**d**) 500 °C, (**e**) 600 °C, and (**f**) 650 °C; Ti is on the left, and Al is on the right.

Figure 9 shows the fracture surfaces after the tensile tests for as-rolled and post-heat-treated clad materials. In Figure 9a, Ti and Al are separated owing to delamination at the Ti/Al interface. The delamination of various bonded materials during tensile testing has been frequently reported in the literature [39]. This phenomenon is closely related to the different plastic strains in each material under the applied stress, and the insufficient diffusion bonding strength produced by the cladding process. In addition, delamination is closely related to the mechanical behavior of the cladding material. Commonly, when delamination occurs at the jointed interface, the stress (or load) in the stress–strain curve declines abruptly, and then fracturing is followed by an increase in tensile deformation. As the tensile strain progresses further, a failure of the specimen may occur prematurely because the crack may connect with the delaminated Ti/Al interface. In contrast to the as-rolled Ti/Al specimen, delamination was less frequent after post-heat treatment at 300 °C. The delamination at the Ti/Al interface did not occur when the post-heat treatment was performed at 400 °C or higher, and the elongation at break at this temperature greatly increased (from 17.8 to 36.8%).

In Figure 9 (the left side is Ti, and the right side is Al), a change in the morphology of the fracture surface can be observed at 400 °C. As mentioned earlier, a high work-hardening effect is induced in the as-rolled specimen, resulting in an increase in strength but a decrease in the ductility of Ti and Al. As shown in Figure 9a, the as-rolled specimen exhibits less ductile fracture, and dimples are hardly observed in both the Al and Ti fracture surfaces. At the same time, with an increase in the post-heat treatment temperature, more dimples are observed on the fracture surface because of an increase in the softening of both Al and Ti metals.

To characterize the mechanical properties at the bonding interface, nanoindentation tests were performed, and the results are listed in Table 4. In the as-rolled specimen, the measured nanohardness and elastic modulus are *H*_Ti_ = 2.41 ± 0.15 GPa and *E*_Ti_ = 118.79 ± 2.88 GPa for Ti and *H*_Al_ = 0.71 ± 0.03 GPa and *E*_Al_ = 83.99 ± 2.10 GPa for Al, respectively, which is consistent with the indentation properties of commercial-purity Ti Grade II [43] and Al 1050 [44]. After the post-heat treatment of the as-rolled specimen, the nanohardness and modulus values of Ti did not vary significantly because the post-heat treatment temperatures were not high enough to induce a change in the microstructural and mechanical properties. In contrast, while the modulus of Al remained almost constant, its hardness continuously decreased with increasing post-heat treatment temperature. The highest Al nanohardness was observed in the as-rolled specimen, because the rolling process increases the dislocation density and refines the grains [45]. However, because the annealing temperature of the Al 1050 alloy is 345 °C [46], the nanohardness of Al decreases, owing to the recovery, recrystallization, and grain growth during the post-heat treatment of the as-rolled Ti/Al material.

**Figure 10:**
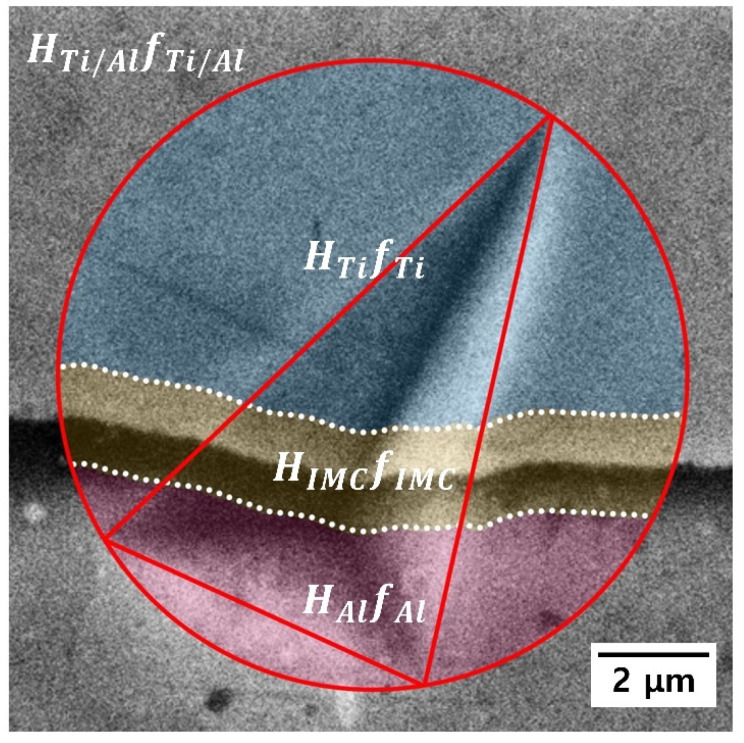
Scanning electron micrograph taken after the nanoindentation test on the Ti/Al-clad material, which includes the Ti/Al interface.

Despite a decrease in the nanohardness of Al, the measured nanohardness at the Ti/Al interface (*H*_Ti/Al_) remained constant until the post-heat treatment temperature of 500 °C, and increased rapidly after the post-heat treatment at 600 and 650 °C. This tendency was attributed to the formation of the intermetallic compound at the Ti/Al interface, as shown in Figure 3e,f. Therefore, to evaluate the effect of the intermetallic compound on the increase in hardness at the interface, the hardness of the bonding interface (*H*_IMC_) was quantitatively determined. *H*_IMC_ may be determined using a simple rule of mixtures based on the individual hardness data [47].

(2) $$H_{IMC}=\left\{H_{Ti/Al}-\left(H_{Ti}f_{Ti}+H_{Al}f_{Al}\right)\right\}/f_{IMC}$$

where *H*_*Ti/Al*_ is the nanohardness obtained from a similar maximum indentation depth (approximately 1.61 μm or more), which includes the intermetallic compound layer, and *f* is the volume fraction of each material within the indentation-induced plastic zone. To determine *f*_*Ti*_, *f*_*Al*_, and *f*_*IMC*_ in Equation (2), the area of the indentation-induced plastic zone was assumed to be the same as that of the circle passing through the three angular points of the triangular hardness impression (as shown by the red circle in Figure 10). The values of *f*_*Ti*_, *f*_*Al*_, and *f*_*IMC*_ were then determined using an image analyzer. The estimated *H*_*IMC*_ was 16.11 ± 4.85 GPa, which is a relatively high value compared to those of Ti and Al. Such a high value was attributed to the formation of the intermetallic compound layer (Figure 3e,f) during the post-heat treatment at 600 and 650 °C, because intermetallic compounds typically exhibit high strength but low ductility. In addition, with increasing post-heat treatment temperature, the intermetallic compound layer became thicker, and *H*_*Ti/Al*_ increased. However, the intermetallic compound that formed at the Ti/Al interface may act as a weak point and a stress concentration area under an external load, thereby reducing the strength of the Ti/Al-clad material, as shown in the tensile test results (Figure 8a).

## 4. Conclusions

In this study, post-heat treatment was performed to improve the mechanical reliability of the Ti/Al-clad material, and the relationship between the microstructure and mechanical properties at the interface of the Ti/Al-clad material was analyzed.

1. Compared to the as-rolled specimen, no significant microstructural differences were observed after the post-heat treatment at 300, 400, or 500 °C. However, after the post-heat treatment at 600 °C and beyond, a new phase layer was formed between Ti and Al. The thickness of this layer increased with post-heat treatment temperature owing to the active diffusion of Al atoms. XRD analysis shows that this layer consisted of four different intermetallic compounds.
2. With an increasing heat treatment temperature, the elongation at break increased, but the strength decreased because of the recovery of Al, which occupied a relatively large area of the cross-section. However, after the post-heat treatment at 600 and 650 °C, the strength remained constant, but the elongation at break decreased because of the growth of the intermetallic compound at the Ti/Al interface, which could act as a stress concentration area.
3. Post-heat treatment decreased the nanohardness of Al owing to recovery, recrystallization, and grain growth; at the same time, the nanohardness of Ti was not affected. The nanohardness of the intermetallic compound at the Ti/Al interface was estimated to be 16.11 ± 4.85 GPa using a simple rule of mixture. Thus, a rapid increase in *H*_Ti/Al_ at the post-heat treatment temperature of 600 °C and higher was attributed to the formation of intermetallic compounds.

## Figures and Tables

**Table 1:** Chemical composition of Ti Grade II and Al 1050.

Sample			Elements (wt. %)									
	H	C	O	N	Al	Ti	Mg	Mn	Fe	Cu	Si	Zn
Ti Gr. Ⅱ	0.015	0.08	0.25	0.03	-	Bal.		-	0.30	-		-
Al 1050	-	-	-	-	Bal.	≤0.03	≤0.05	≤0.05	≤0.40	≤0.05	≤0.25	≤0.05

**Table 2:** Coefficients of Al-in-Ti and Ti-in-Al diffusions.

	Diffusion of Al in Ti		Diffusion of Ti in Al		
Temp. Range (℃)	$D_{0}$ (m^2^/s)	*Q* (J/mol)	$D_{0}$ (m^2^/s)	*Q* (J/mol)	Refs.
300–450	8.32 × 10^−6^	159,275	1.12 × 10^−1^	260,000	[24,25,26]
530–600	2.28 × 10^−7^	105,100	1.12 × 10^−1^	260,000	[24,25,27]

**Table 3:** Room temperature tensile properties of specimens post-heat-treated at different temperatures.

	UTS (MPa)	YS (MPa)	EL (%)
As-rolled	166.0 ± 2.01	139.6 ± 4.23	11.4 ± 0.87
300 °C	152.6 ± 6.12	131.6 ± 5.32	17.8 ± 4.91
400 °C	128.4 ± 1.33	97.0 ± 3.69	36.8 ± 0.88
500 °C	122.1 ± 1.72	86.6 ± 2.42	38.0 ± 0.56
600 °C	115.5 ± 1.42	74.7 ± 2.04	43.6 ± 1.82
650 °C	120.3 ± 2.41	75.1 ± 0.29	43.1 ± 1.13

**Table 4:** Nanohardness and modulus of Ti, Al, and the Ti/Al interface of the as-rolled and post-heat-treated Ti/Al-clad materials.

Specimen	*H*_Ti_ (GPa)	*E*_Ti_ (GPa)	*H*_Al_ (GPa)	*E*_Al_ (GPa)	*H*_Ti/Al_ (GPa)
As-Rolled	2.41 ± 0.15	118.79 ± 2.88	0.71 ± 0.03	83.99 ± 2.10	3.92 ± 0.52
300 °C	2.71 ± 0.18	122.72 ± 3.16	0.65 ± 0.05	82.08 ± 2.18	3.29 ± 0.40
400 °C	2.54 ± 0.21	119.60 ± 2.92	0.61 ± 0.03	79.74 ± 1.83	3.88 ± 0.61
500 °C	2.78 ± 0.20	122.45 ± 3.24	0.59 ± 0.06	78.61 ± 3.33	3.00 ± 0.39
600 °C	2.36 ± 0.16	122.61 ± 3.84	0.58 ± 0.03	79.86 ± 1.45	4.74 ± 0.71
650 °C	2.66 ± 0.34	122.21 ± 4.50	0.58 ± 0.02	80.92 ± 1.25	5.08 ± 0.60

## Data Availability

Not applicable.
